# Molecular Mechanisms Regulating Temperature Compensation of the Circadian Clock

**DOI:** 10.3389/fneur.2017.00161

**Published:** 2017-04-27

**Authors:** Rajesh Narasimamurthy, David M. Virshup

**Affiliations:** ^1^Program in Cancer and Stem Cell Biology, Duke-NUS Medical School, Singapore, Singapore

**Keywords:** circadian clock, temperature compensation, phosphorylation, phosphoswitch, period2

## Abstract

An approximately 24-h biological timekeeping mechanism called the circadian clock is present in virtually all light-sensitive organisms from cyanobacteria to humans. The clock system regulates our sleep–wake cycle, feeding–fasting, hormonal secretion, body temperature, and many other physiological functions. Signals from the master circadian oscillator entrain peripheral clocks using a variety of neural and hormonal signals. Even centrally controlled internal temperature fluctuations can entrain the peripheral circadian clocks. But, unlike other chemical reactions, the output of the clock system remains nearly constant with fluctuations in ambient temperature, a phenomenon known as temperature compensation. In this brief review, we focus on recent advances in our understanding of the posttranslational modifications, especially a phosphoswitch mechanism controlling the stability of PER2 and its implications for the regulation of temperature compensation.

The main advantage of having an intact circadian clock system is to anticipate and alert our physiological mechanisms to prepare for daily changes in the environment imposed by light–dark cycle of the earth. At the organism level, the circadian clock is a hierarchical multioscillator network, where in mammals, the suprachiasmatic nuclei (SCN) is the master oscillator. The SCN in the hypothalamus of brain is entrained by the light–dark cycle through the eye and neuronal retinal ganglion cells. Synchronized highly interconnected neurons in the SCN oscillate and transmit their rhythm to peripheral oscillators such as liver, lung, and kidney *via* systemic cues including neuronal, neuroendocrine, and behavioral pathways. This clock network entrains physiological processes including the sleep–wake cycle, liver metabolism, and body temperature (1–3). At the molecular level, the circadian clock is composed of transcriptional and translational feedback loops that oscillate in cycles of approximately 24-h to create the circadian rhythms we see at the organism level. In the core loop, the positive transcriptional activators Clock and Bmal1 bind to E-box motifs and activate the expression of many targets, including their own negative regulators, Period (Per1, 2, and 3) and Cryptochromes (Cry1 and Cry2). As the negative feedback proteins Per and Cry increase in abundance, they multimerize, enter into the nucleus, and bind to the heterodimeric Clock and Bmal1 complex to inhibit their transcriptional activity. This generates a 24-h cycle that is cell autonomous. This clock machinery is broadly functional in all mammalian tissues (1–3).

The three major hallmarks of circadian clocks are their ~24-h oscillation in the absence of any external stimuli, entrainment by external stimuli, and temperature compensation. Entrainment allows the master clock to synchronize with seasonally and geographically changing light–dark cycles. In mammals, light entrains the central clock *via* retinal ganglion cells that communicate with the SCN *via* the retinal–hypothalamic tract. Homeothermic animals such as mammals maintain a nearly constant body temperature with a narrow range of fluctuations in most part of the body, whereas poikilotherms such as frogs have body temperature, which can vary in wide range (4, 5). However, even in mammals, peripheral clocks can be entrained by small daily oscillations in internal body temperature (1, 3, 6).

Although the circadian clock system can be entrained by fluctuations in temperature, it remains fairly resistant to ambient temperature-induced changes in circadian period (5, 7). According to the Arrhenius equation of temperature dependence on reaction rate, in any (bio)chemical reaction, a rise in temperature increases the rate of the reaction (8), which eventually reduces the reaction time. But in the case of the circadian biochemical system, in spite of changes in ambient temperature, the period length remains essentially constant at approximately 24-h. Thus, Pittendrigh demonstrated that the *Drosophila* rhythm of eclosion (emergence of the adult fly from the pupa) retained a 24-h rhythmicity in total darkness over a temperature range of 16–26°C (5). This phenomenon is referred to as temperature compensation (5, 9). The temperature compensation of circadian period is evolutionarily conserved from light-sensitive cyanobacteria to homeothermic mammals, and surprisingly, even an *in vitro* circadian clock reconstituted with KaiABC proteins of cyanobacteria shows temperature compensation between 25 and 35°C, suggesting that it is a core design feature of the molecular clock (5, 10–13). More recently, using tissue explants and cell culture, it has been demonstrated that temperature compensation is a tissue and cell autonomous property. For example, the circadian oscillators controlling melatonin synthesis in the retina of golden hamsters are temperature compensated between 27 and 33°C (14), and *Per1^Luc^* fibroblasts maintain ~24-h period length despite changes in temperature over the range of 28.5–36.5°C (12). These findings also confirm peripheral cells as *bona fide* model systems to study the temperature compensation mechanism of the circadian clock (12).

## Models of Temperature Compensation

How the active process of temperature compensation is achieved by organisms is an area of intense research interest to both chronobiologists and mathematical modelers. Hastings and Sweeney almost 60 years ago proposed that temperature compensation could be achieved if two temperature-dependent reactions oppose each other, although at the time there was no inkling of what those reactions might be (9). This conceptual model was extended by Ruoff with the notion that positive and negative feedback loops of the oscillators might act as the opposing reactions and lead to temperature compensation in any kinetic oscillator model (15). As specific molecular members of the clock were identified, Hong et al. first proposed that PER protein dimerization might regulate temperature compensation (16). Ten years later, as the complexity of the clock mechanism became clearer, many of the newly described regulatory steps have been tested in mathematical models of the clock to assess their potential contribution to temperature compensation. For example, Hong et al. suggested that switch-like mechanisms acting on sensitive parameters such as phosphorylation, ubiquitination, or complex formation controlling PER protein might regulate temperature compensation (17). Others have suggested using modeling that the concentration of a rate-limiting enzyme involved in processes like phosphorylation can determine temperature compensation (18).

In *Neurospora*, the core clock gene *frequency* (*frq*) undergoes alternative splicing that is temperature sensitive. The resulting two isoforms have opposing effects on clock speed and was once proposed to underlie temperature compensation (19–21). More recently, casein kinase 2 in *Neurospora* was implicated in temperature compensation. Decreased CK2 activity, or mutation of a specific CK2 phosphorylation site, leads to altered temperature compensation, probably due to an altered balance of phosphorylation at distinct sites. Interestingly, CK2 itself had a normal Q10, i.e., its activity changed twofold with a 10°C increase in temperature (22). In this system, casein kinase 1 (CK1) was important for clock speed but not temperature compensation. Although these studies have provided some insights for understanding the mechanisms of temperature compensation, either they lack good experimental evidence to support their mathematical model or these models are not tested in mammalian system.

## Phosphorylation of PER2 Controls Clock Speed

**Figure 1:**
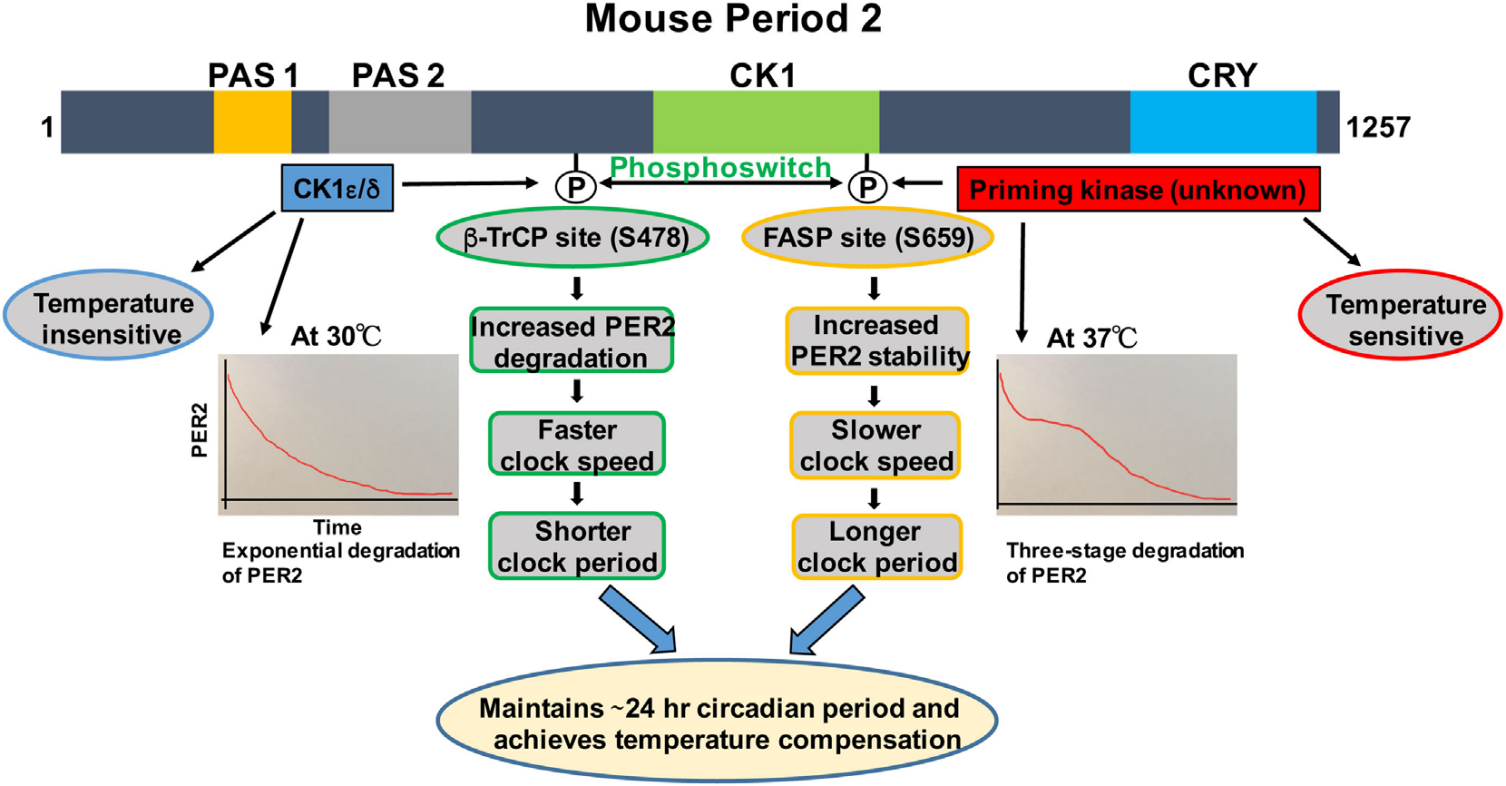
**Regulation of PER2 phosphorylation, degradation, and its role in temperature compensation by the phosphoswitch mechanism**. Lower temperature increases relative phosphorylation at the β-TrCP site of PER2, leading to faster degradation and shorter period. Higher temperature increases relative familial advanced sleep phase (FASP) site phosphorylation, enhancing PER2 stability and lengthening the period. The degradation pattern of PER2 at 30°C is largely exponential, while at 37°C, three-phase degradation is seen. This has important implications for temperature compensation (see text for details). Domain architectures are shown in colors. PAS1, PAS domain 1 (orange); PAS 2, PAS domain 2 (grey); CK1, Casein kinase 1-binding domain (green); CRY, Cry binding site (blue).

Many of the mathematical models suggested that temperature compensation could be due to two opposing reactions acting on a rate-limiting step of the circadian clock machinery (9, 18). The reversible multisite phosphorylation of PER2 is a potential target in this regard due to its rate-limiting role in regulating clock speed (Figure 1) (23, 24). The importance of phosphorylation in the control of circadian rhythms was demonstrated first by the finding of short- and long-period mutations in *Drosophila* that both mapped to the Dbt kinase gene, the ortholog of mammalian CK1δ and CK1ε (25, 26). CK1 is a family of serine/threonine kinases with seven different isoforms in mammals that are encoded by distinct genes (α, β, γ1, γ2, γ3, δ, and ε), which are involved in diverse biological functions including circadian rhythms, Wnt signaling, membrane trafficking, cytoskeleton maintenance, DNA replication, DNA damage response, RNA metabolism, and parasitic infections (23, 27–30). The first circadian clock phenotype in mammals was found in *tau* hamsters with 20-h short period (31). Later, it was identified that a missense mutation in hamster CK1ε^tau^(Arg178Cys) is to underlie the short-period phenotype of the *tau* hamster (32). Subsequently, point mutation of a CK1δ/ε-regulated motif in human PER2 [S662G, familial advanced sleep phase (FASP) site] (33) and a point mutation of CK1δ were found in families with FASP syndrome (34). A body of evidence suggests that CK1δ is the major driver of clock timing, but that CK1ε plays an important role as well.

The mechanism by which CK1 regulates phosphorylation of PER2 is complex and is slowly being teased apart. Phosphorylation of PER2 by CK1ε leads to recruitment of the ubiquitin ligase, β-TrCP, and proteasomal-mediated degradation of PER2 (35). But the impact of CK1ε activity on the clock speed has been puzzling, due to opposing observations that reduced CK1 activity shorten (32, 34) and lengthen the circadian period (35). To solve this puzzle, mathematical modeling was applied and then experimentally confirmed the non-intuitive prediction that the short-period *tau* mutation of CK1ε is in fact functionally a gain of function, not a loss of function mutation. It was further reported that the CK1ε^tau^ is a highly specific gain of function for its substrate PER2, which gets phosphorylated and degraded much faster, resulting in a faster clock and shorter circadian period (36). These studies emphasized the value of combining experimental studies with predictive mathematical models to advance our understanding of the clock and how changes in kinase activity can alter the clock.

## A Phosphoswitch Regulates PER2 Degradation

We and others have shown that there are two phosphorylation sites, the FASP and the β-TrCP site, regulating stability of mammalian PER2 (Figure 1) (35, 37). The FASP site is a missense mutation at S662G (S659 in mouse) associated with FASPS, which prevents priming phosphorylation by an unknown priming kinase. Priming phosphorylation of S659 (FASP site) is required for the phosphorylation of four immediate downstream serines of PER2 (659-**S**VVSLTSQCSYSS-671) by CK1ε/δ (33, 37). The second functional phosphorylation site is β-TrCP site that is also a CK1ε-dependent phosphorylation site (S478 in mPER2), but that seems to be independent of priming phosphorylation (35). It has been identified that surprisingly PER2 undergoes three distinct stages of degradation upon addition of the protein synthesis inhibitor cycloheximide during the PER2 accumulation phase (CT 14–26) of the circadian cycle. Mathematical modeling predicts that a phosphoswitch generates the three-stage degradation of PER2 (38). Accordingly, the first rapid decay phase is β-TrCP site phosphorylation dependent, the second slow plateau phase is dependent on FASP site phosphorylation, and in the third and falling phase, PER2 protein is degraded in a CK1δ/ε-independent manner that is not well understood. Importantly, the model was experimentally confirmed (38). Further experiments showed that CK1ε^tau^ has decreased activity on the FASP site, leading to an increased activity on the β-TrCP (S478) site. This explains how CK1ε^tau^ is a gain of function on phosphorylation at S478 and further supports the phosphoswitch between the two sites (the FASP and the β-TrCP site) (38).

## Period2 Phosphoswitch Unravels the Mechanism of Temperature Compensation

Before CK1ε was even identified as a clock component, its role in temperature compensation was suggested by the observation that retinas from *tau* mutant hamsters have significantly impaired temperature compensation (14). Isojima et al. subsequently reported that unlike virtually all other kinases, CK1ε/δ are temperature insensitive (39). Therefore, they proposed that CK1ε/δ-dependent phosphorylation process might play a central role in temperature compensation of the circadian clock (39). Indeed, in further study, the CK1ε/δ phosphorylation of a β-TrCP peptide was temperature insensitive (39). The mathematical model of Kim and Forger, building on the pioneering work of Forger and Peskin in understanding the mammalian clock system using mathematical tools (40–42), predicted a potential role for the phosphoswitch mechanism in temperature compensation. A key feature of the model requires that there are two sites involved in the phosphoswitch, the FASP and the β-TrCP sites (Figure 1) (38). Since CK1 is relatively temperature insensitive (39), the model assume that priming of the FASP site has normal temperature sensitivity, i.e., its activity increases with increasing temperature, while CK1ε/δ phosphorylation of the β-TrCP site is temperature insensitive, i.e., the rate of phosphorylation is constant regardless of temperature. Incorporating this differential kinase temperature sensitivity into the mathematical model indeed predicted that this could underlie temperature compensation. This model was then experimentally tested in immortalized *Per2^Luc^* mouse embryonic fibroblasts (MEFs). It was found that at higher temperatures, increased FASP site phosphorylation by the priming kinase leads to slow second-phase degradation and more accumulation of PER2, eventually lengthening and compensating period length. Similarly, *Per2^Luc^* MEFs at 30°C showed a marked decrease in second-phase degradation, whereas first-stage degradation remained intact. These findings underscore the importance of the relative rates of phosphorylation of the two phosphoswitch sites in temperature compensation (38). Additional experiments indirectly tested if an intact phosphoswitch mechanism is necessary for temperature overcompensation. An abnormal temperature compensation was observed in CK1ε^tau^; *Per2^Luc^* MEFs, and also in *Per2^Luc^* MEFs treated with a CK1ε/δ inhibitor, further supporting a role for CK1ε/δ and an intact phosphoswitch mechanism as a prerequisite for temperature compensation. The studies also support the value of a robust mathematical model that makes testable predictions about complex systems when biological intuition has reached its limits (38).

Recently, it has been reported that cells with knockouts of specific circadian clock components retain temperature compensation (43). The authors concluded that temperature compensation is likely determined by a rate-limiting process(es) that are temperature sensitive, consistent with the phosphoswitch mechanism (43). Another mathematical model for temperature compensation has recently proposed a temperature insulation mechanism where oscillation period is determined by very few temperature-independent or only slightly temperature-dependent parameters, but where other parameters remain strongly temperature dependent (44). This model is analogous to the proposed phosphoswitch mechanism in which the CK1ε/δ is temperature independent or slightly dependent, whereas the priming kinase is temperature dependent (38).

There are a number of unresolved issues. The priming kinase has not been identified yet. It also remains unclear what happens to PER2 phosphorylation over the full 24-h day, in part because the methods to study this in mammalian systems are not suitably sensitive. This is relevant to another unsolved question: how PER2 is degraded in the third phase of three phase decay, when neither CK1 nor proteasome inhibitors impact PER2 loss? Moreover, further study is necessary to understand whether fluctuations in body temperature, which can entrain the clock, do so in part *via* the phosphoswitch mechanism in addition to the proposed heat shock factor 1 (HSF1) mechanism (7, 45). Finally, it is also important to address whether the mechanisms regulating temperature compensation in peripheral cells and the central pacemaker (SCN) cells are the same and whether temperature-induced changes in peripheral clocks can feed back to the central clock.

## The Outlook

It is remarkable that the complex yet robust phenomenon of temperature compensation is regulated by subtle differences in phosphorylation of the same protein at different sites. Notably, this finding is in general agreement with predictions of earlier mathematical models that suggested that opposing outputs with switch-like mechanisms might control temperature compensation (9, 17). In the future, it will be important to identify the priming kinase that plays a central role in the phosphoswitch model. This phosphoswitch mechanism of temperature compensation may be a core feature of clocks in many species, as a similar interaction of phosphorylation sites is operative in *Drosophila* and *Neurospora* as well (46–48).

## Author Contributions

RN and DV were involved in conceptualizing, researching and writing this review.

## Conflict of Interest Statement

The authors declare that the research was conducted in the absence of any commercial or financial relationships that could be construed as a potential conflict of interest.
